# Electrospun Smart Hybrid Nanofibers for Multifaceted Applications

**DOI:** 10.1002/marc.202400617

**Published:** 2024-10-14

**Authors:** Viraj P. Nirwan, Altangerel Amarjargal, Rebecca Hengsbach, Amir Fahmi

**Affiliations:** ^1^ Faculty of Technology and Bionics Rhine‐Waal University of Applied Science Marie‐Curie‐Straße 1 47533 Kleve Germany; ^2^ Power Engineering School Mongolian University of Science and Technology 8th khoroo, Baga toiruu, Sukhbaatar district Ulaanbaatar 14191 Mongolia

**Keywords:** biomedicine, nanocatalysis, nanoenergy generation, Smart nanomaterials, targeted drug delivery

## Abstract

Smart electrospun hybrid nanofibers represent a cutting‐edge class of functional nanostructured materials with unique collective properties. This review aims to provide a comprehensive overview of the applications of smart electrospun hybrid nanofibers in the fields of energy, catalysis, and biomedicine. Electrospinning is a powerful tool to fabricate different types of nanofibers’ morphologies with precise control over structure and compositions. Through the incorporation of various functional components, such as nanoparticles, nanomoieties, and biomolecules, into the (co)polymer matrix, nanofibers can be tailored into smart hybrid materials exhibiting responsiveness to external stimuli such as temperature, pH, or light among others. Herein recent advancements in fabrication strategies for electrospun smart hybrid nanofibers are discussed, focusing on different electrospinning tools aimed at tailoring and developing smart hybrid nanofibers. These strategies include surface functionalization, doping, and templating, which enable fine‐tuning of mechanical strength, conductivity, and biocompatibility. The review explores the challenges and recent progress in the development of smart hybrid nanofibers. Issues such as scalability, reproducibility, biocompatibility, and environmental sustainability are identified as key for improvement. Furthermore, the applications of smart nanofibers in biomedicine, environment, energy storage, and smart textiles underscore their potential to address the challenges in development of nanostructured materials for emerging technologies.

## Graphical abstract

The review provides a comprehensive overview of the applications of smart electrospun hybrid nanofibers in the fields of energy, catalysis, and biomedicine, highlighting their remarkable properties for diverse applications. Exploration of the challenges and recent progress in the development of smart hybrid nanofibers is conducted. Issues such as scalability, reproducibility, biocompatibility, and environmental sustainability are identified as key areas for improvement.

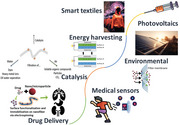

## 1 Introduction

**Figure 1:**
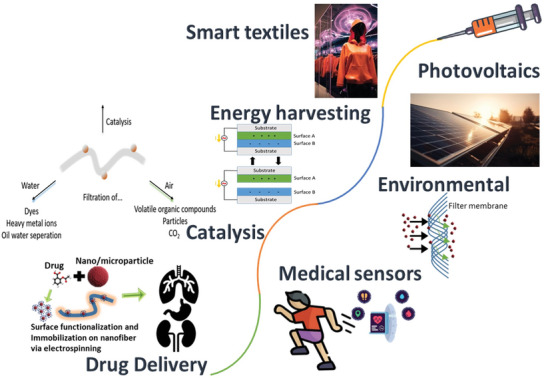
Multifaceted applications arising from hybrid nanofibers.

Advanced nanostructured hybrid materials, particularly electrospun hybrid nanofibers, represent the progress of innovation in materials science and engineering. These hybrid materials can be referred to as unidirectional structured materials engineered at the nanoscale using chemistry and electrospinning techniques. Electrospinning enables the fabrication of fibers on the nanoscale with precise control over composition, morphology, and structure.^[^ ^1^ ^]^ Electrospun hybrid nanofibers offer many advantages and functionalities that transcend traditional structured materials due to their unique collective properties (**Figure** **1**). The versatility and tunability of electrospun hybrid nanofibers facilitate vast numbers of functional materials that possess unique transformative properties across different industrial sectors.^[^ ^2^ ^]^

Electrospinning involves the use of electric potential to draw charged polymer solutions or melts into ultrafine fibers.^[^ ^3^, ^4^, ^5^ ^]^ This process can be further optimized and the generated nanofibers functionalized by incorporating various nanomaterials, such as nanoparticles, nanotubes, or nanosheets, into the polymer matrix. The resulting hybrid nanofibers exhibit synergistic tailored properties, including high surface area, tunable porosity, mechanical flexibility, electrical conductivity, and biocompatibility. These are also driven by the combination of different components to open up new avenues for innovation and shaping technological advancements in healthcare, environmental sustainability, energy, textiles, and materials science^[^ ^5^, ^6^, ^7^ ^]^ (Figure 1). As research and development in this field continue to progress, the potential for further innovation and impact is immense, promising a future with smarter, more sustainable, and more efficient solutions for a wide range of applications.^[^ ^8^, ^9^ ^]^ In this context, investigations have been performed to understand the possibilities of using electrospun hybrid nanofibers and uncovering their significant role in the catalysis field for innovation driving progress in materials science and engineering.^[^ ^10^ ^]^ In biomedicine, these nanofibers can be tailored to mimic the extracellular matrix, offering promising prospects for tissue engineering, drug delivery, and wound healing.^[^ ^6^, ^11^, ^12^, ^13^, ^14^ ^]^ In environmental applications, electrospun hybrid nanofibers have shown great potential as filtration membranes for water purification, air filtration, and oil spill cleanup.^[^ ^15^ ^]^ Moreover, their use in energy‐related technologies, such as electrodes for batteries and supercapacitors, highlights their significance in advancing sustainable energy solutions.^[^ ^16^ ^]^

This review aims to delve into the fundamental principles of smart hybrid nanofiber synthesis, characterization, and manipulation, shedding light on their remarkable properties and potential applications. Through an extensive exploration proposed to provide insights into the smart nanofibers' latest advancements, challenges, and future directions in this rapidly evolving domain including biomedicine and environmental remediation to energy storage and beyond.

## 2 Control Over the Electrospun Hybrid Nanofiber Morphologies

The electrospinning technique is a method that utilizes electrostatic forces to create continuous nanofibers. The process involves loading a polymeric solution or melt into a syringe or reservoir, which is then subjected to high voltage to create an electric field. This generates a fine jet through a small nozzle, which solidifies into nanofibers as the solvent evaporates. As electrospinning employs high voltage, it requires overcoming surface tension, considering the solvent's dielectric property, adjusting solution parameters, processing parameters, and ambient parameters, selecting the appropriate collector type, and choosing suitable materials. All these factors work together to control the morphology, diameter, and properties of the electrospun nanofibers.

### 2.1 Solution Properties

The properties of the polymer solution or melt, including viscosity, surface tension, and conductivity, significantly influence fiber morphology. Adjusting parameters such as polymer concentration, solvent type, and additive incorporation can alter these properties to achieve desired fiber characteristics. To produce fibers with the desired diameter and mechanical strength, it is crucial to consider the polymer concentration and viscosity in the solution. Increasing these factors can result in thicker and sturdier fibers. Another significant aspect to consider in the electrospinning process is the solution conductivity, which can affect the formation and stability of the jet. Opting for a solution with higher conductivity can lead to more consistent and uniform fibers. Additionally, the choice of solvent used in the polymer solution plays a critical role since different solvents can affect the polymer's solubility, viscosity, and evaporation rate, ultimately impacting the fiber morphology and properties.^[^ ^17^, ^18^, ^19^, ^20^, ^21^, ^22^ ^]^

### 2.2 Process Parameters

These are due to the changes in the electrospinning parameters including flow rate, distance, collector speed, and voltage. For instance, the voltage affects fiber diameter and morphology. Higher voltages lead to thinner fibers due to increased stretching forces. The flow rate influences fiber diameter and uniformity. Higher flow rates typically result in thicker fibers. The distance between the spinneret and collector is inflecting the fiber stretching and alignment, for example, longer distances can lead to more aligned fibers. The collector speed can affect fiber alignment and deposition pattern.^[^ ^23^, ^24^, ^25^ ^]^ The conditions surrounding the electrospinning process are known as the ambient conditions, which include variables such as temperature and relative humidity. The solvent evaporation rate is directly impacted by these variables, which ultimately determines the outcome of jet solidification. Insufficient humidity levels can result in thin fibers with a dry surface, while excessively low levels may hinder the jet extension process. On the other hand, high humidity can cause significant morphological changes in nanofibers. Changes in temperature have a noticeable effect on the average diameter of nanofibers. Higher temperatures result in faster solvent evaporation, altering the size of the nanofibers, while lower temperatures decrease the viscosity of the solution, which also impacts the average diameter of the nanofibers.^[^ ^26^, ^27^, ^28^ ^]^

### 2.3 Coaxial, Janus, and Emulsion Electrospinning

**Figure 2:**
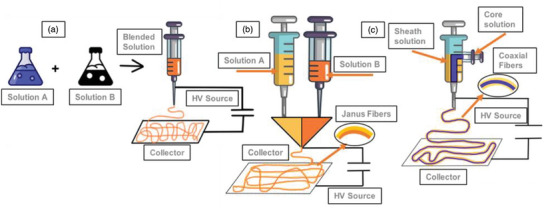
Electrospinning of a) Blending, b) Janus, and c) Coaxial structures. Influencing the setup, parameters, and compositions during electrospinning offers state of the art properties in nanofibers.

The electrospinning of multifluid as well as colloids represents a flourishing field with the potential to produce sophisticated multicompartment nanofibers through a variety of techniques for organizing the inner components, using both spinnable and unspinnable working fluids.^[^ ^29^, ^30^, ^31^, ^32^ ^]^ This presents a distinctive chance to create extra features or enhance the performance of fibers that are different from those produced by mono‐axial spinning. These techniques involve using coaxial, side‐by‐side, and emulsion setups to electrospin core‐shell, Janus, or composite fibers, enabling precise control over fiber morphology and functionality (**Figure** **2**). Moreover, incorporating additives such as surfactants, cross‐linkers, or functional nanoparticles into the polymer solution can modify fiber morphology, alignment, and properties. Unlike colloid electrospinning, multifluid electrospinning demands a more coordinated approach to ensure success, including precise attention to the spinneret, the choice of working fluids, and operational conditions.^[^ ^33^ ^]^ Although the side‐by‐side electrospinning technique is widely considered a challenging technology compared to the coaxial approach, it offers several advantages due to the ability to create bi‐compartmented constructions with multiple domains of independent properties. Specifically, the Janus fiber has been shown to be advantageous over core‐shell nanofibers, which entail complete coverage of the interior by the exterior part. With Janus fiber, both regions can make direct contact with the surrounding medium, enabling the creation of a dual‐responsive material that can be tailored to respond to specific stimuli. The proper arrangement of the needle plays a crucial role in creating Janus nanofibers using a side‐by‐side electrospinning system.^[^ ^34^ ^]^ Using a two‐needle spinneret often leads to failed attempts to produce integrated Janus structures due to the identical charges of the two fluids, which may have separated at the beginning of the spinning process. To ensure the production of high‐quality Janus nanofibers, it is necessary to use a spinneret that maximizes the contact area between the spinning solutions.^[^ ^35^, ^36^, ^37^ ^]^ One way to achieve this is by modifying the dual metal capillaries that attach a plastic pipe to its end, which increases the contact surface of the polymer solution's two sides and provides prolonged contact time.^[^ ^38^ ^]^ Additionally, dissolving both spinning polymers in the same solvent could enable precise control over the movement of the two solutions in unison, leading to the accurate formation of a Janus Taylor cone.^[^ ^39^ ^]^ The simple change of spinneret and the same solvent system of two spinning solutions helped achieve exceptional results and ensured the highly yielded anisotropic Janus structure with two faces. On the other hand, various post‐treatment methods, including heat treatment, chemical treatment, and surface modification, can further tune nanofiber morphology, porosity, and surface chemistry.

## 3 Multifaceted Application of Smart Hybrid Nanofibers

### 3.1 Smart Textiles as Wearable Technology and Integrated Smart Components

Electrospun nanofibers are playing an important role in textile technology. In particular, advancements in electrospun hybrid nanofibers with integrating functionalities are driving innovation in smart textiles. Their unique collective properties, biocompatibility, sensitivity, and flexibility make them an ideal platform for developing the next generation of devices across various applications from healthcare,^[^ ^40^ ^]^ environmental monitoring,^[^ ^41^, ^42^ ^]^ energy harvesting,^[^ ^43^, ^44^ ^]^ and wearable electronics with enhanced functionalities, performance, comfort, and sustainability.^[^ ^45^ ^]^

**Figure 3:**
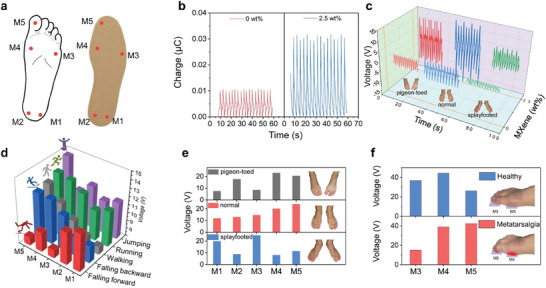
Scheme of insole with five integrated piezoelectric textiles as sensor network a), with the piezoelectric charges of undoped fibers and MXene doped fibers induced by toe pressing of a 75 kg adult b), the output voltages depending on the posture c) and the signal profiles for gait monitoring d), posture recognition e) as well as Metatarsalgia prognosis f). Reproduced (Adapted) with permission.^[^ ^55^ ^]^ Copyright 2024, ACS Publications.

Smart textiles known as smart fabrics or e‐textiles are based on smart hybrid nanofibers and refer to fabrics that incorporate responsive compartments via additive technologies to provide further functionalities beyond traditional textiles. These textiles can be integrated or designed as wearables or can be applied to other existing modules within automobiles, filter assemblies, and wound dressing among others to interact with the wearer or the environment, offering features such as sensing,^[^ ^46^ ^]^ actuation,^[^ ^47^ ^]^ communication,^[^ ^48^ ^]^ and energy generation or storage.^[^ ^49^, ^50^ ^]^ Key aspects of smart textiles are multifunctionality where electrospun hybrid nanofibers can be engineered with varieties of functional molecules at nanoscales to possess multiple functionalities tailored simultaneously, such as conductivity, sensitive sensing capabilities, self‐cleaning properties,^[^ ^51^, ^52^ ^]^ and controlled drug release.^[^ ^53^ ^]^ For instance, integrating conductive materials, such as carbon nanotubes, graphene, or metallic nanoparticles, into electrospun nanofibers enables the fabrication of textiles with electronic functionalities.^[^ ^54^ ^]^ This integration often involves innovative fabrication techniques such as weaving, knitting, printing, or coating with conductive materials. The fabricated textiles can be used for wearable electronics, biomedical sensors, and energy‐harvesting devices.^[^ ^55^, ^56^ ^]^ For instance, as seen in **Figure** **3**, sensors can be integrated into the insoles of the shoes to detect walking profiles. Based on the walking profiles recorded through these sensors, it is possible to diagnose posture abnormalities and monitor the gait of the wearer. Moreover, through the data acquired via gait monitoring, it is possible to correlate the changes in voltages and output signal with the prognosis of metatarsalgia, which is a painful inflammation of the ball of the foot.^[^ ^55^ ^]^ Therefore, emerging as a useful tool in preventive medicine. In this context, certain smart textiles incorporate energy harvesting technologies to generate electricity from ambient sources such as motion, light, or heat. They may also include integrated energy storage components such as batteries or supercapacitors, enabling the autonomous operation of embedded electronics. Smart textiles can incorporate responsive materials that change their properties in response to external stimuli. The significant change in properties facilitates sensitive sensing effects to detect various parameters such as temperature,^[^ ^57^ ^]^ humidity,^[^ ^58^ ^]^ pH,^[^ ^59^ ^]^ pressure,^[^ ^60^ ^]^ motion, biometric data (heart rate, respiration rate, etc.)^[^ ^61^ ^]^ and environmental pollutants.^[^ ^62^ ^]^ The fast response rate reflects the sensitivity of the polymer chains due to the application of external stimuli to control the mass/energy transfer diffusion. Once the stimuli‐responsive polymer chains are formed in the nanofibers via electrospinning techniques, the resultant larger specific surface area to volume and the higher porosity can facilitate the high sensitivity. The higher porosity and the larger specific surface area contribute to shorter diffusion distances and more interactive sites, respectively.^[^ ^63^, ^64^ ^]^ Besides, the smart properties can be designed because of the distinctive polymeric architectures, which possess special effects like piezoelectric and triboelectric effects and are considered one of the best options for the fabrication of flexible electronics.^[^ ^65^ ^]^

The highly sensitive sensors can also provide real‐time feedback to the wearer or transmit data to external devices for monitoring and analysis.^[^ ^66^ ^]^ Further, can allow the exchange of data with other devices or networks for safety purposes, or integration with the Internet of Things (IoT) for smart home or healthcare applications.^[^ ^67^ ^]^ Several studies have demonstrated the transformative impact of integrating hybrid nanofibers into smart textiles to augment functionality and performance across various healthy domains. For instance, in a study by Dong et al. hybrid nanofiber‐based wound dressings were developed by incorporating antibacterial agents and growth factors, resulting in enhanced wound healing properties and reduced risk of infection.^[^ ^68^, ^69^ ^]^ Furthermore, the versatility and adaptability of hybrid nanofibers allow for the creation of multifunctional textiles with diverse capabilities. For instance, in a study by Guo et al., hybrid nanofiber membranes were utilized for the simultaneous detection and removal of heavy metal ions from water, displaying the potential of smart textiles for environmental remediation.^[^ ^70^ ^]^ Recently, during the Corona pandemic, the effectivity of hybrid nanofibers for use in the fabrication of masks emphasized its potential infiltration of particulate matter besides pathogens. Thereby, preventing the transfer and spread of potentially hazardous materials. Across the globe, research teams and industries have applied hybrid nanofibers by incorporating particles, which are known for their antibacterial and anti‐microbial activities.^[^ ^71^, ^72^ ^]^ Therefore, unleashing both physical and chemical modes of filtration. Moreover, the flexibility and conformity of the nanofiber scaffolds afforded easy processability and transferability to preexisting mask fabrication machinery. Furthermore, allowing control over porosity, and morphology to control the filtrate size and pressure drop increases their comfortness without compromising on the effectivity.^[^ ^73^, ^74^ ^]^

### 3.2 Energy Harvesting, Conversion, and Storage Application

With global emphasis on the reduction of dependency on fossil fuels and traditional sources of energy^[^ ^75^, ^76^ ^]^ the economies have been restructured to utilize innovative avenues of energy, which are based on the pillars of renewability and sustainability.^[^ ^77^ ^]^ Therefore, the structural and environmentally focused changes have underlined the importance of photovoltaics, hydrogen fuel cells, piezoelectric, and triboelectric energy sources supporting the existing fossil‐independent technologies like hydropower and nuclear.^[^ ^78^, ^79^, ^80^, ^81^ ^]^

Many technical and economic challenges hinder the adoption of these technologies.^[^ ^82^ ^]^ Here, the focus will be on the development of smart hybrid nanofibers for applications in energy harvesting, conversion, and storage technologies. Specifically, in the form of nanofibers which hold significant potential in energy storage applications due to the presence of properties such as high aspect ratio, flexibility, and tunable morphology. Moreover, the use of electrospinning provides a selection of a combination of materials ideal for such applications. Nanofibers when immobilized with various inorganic functional agents have shown an improvement in the light absorption properties of solar cells. Masoud Abrari et al. in their recent study have focused on Zirconium oxide(ZrO_2_) nanofibers in zinc oxide (ZnO)‐based photoanodes to increase dye‐sensitized solar cells (DSSCs) efficiency. Using the electron‐conducting properties of ZrO_2_ and ZnO and the flexibility of electrospun polyvinylpyrrolidone (PVP) nanofibers, they fabricated a photoanode after calcinating the polymer.^[^ ^83^ ^]^ These photoanodes were used to prepare solar cells, which showed a 97% improvement in efficiency due to increased charge transfer. Interestingly, the efficiency was found to be decreased beyond a certain concentration of inorganic materials.^[^ ^83^ ^]^ Similarly, in another study by Yu‐Hsun Nien et al. on DSSCs with the implementation of Fe_2_O_3_/g‐C_3_N_4_/TiO_2_ heterogeneous nanofibers an increased conversion efficiency of up to 4.81% has been reported, which is 20.85% higher than the values obtained using pure titanium oxide (TiO_2_) photoanode. Moreover, at a low intensity of 30 mW cm^−2^, the highest conversion efficiency of 6.81% was reported.^[^ ^84^ ^]^ These processes show an improvement in the solar conversion efficiency by the use of inorganic functional materials however, the approach in these strategies requires multiple processing steps that have their challenges in terms of reproducibility, average photoconversion efficiencies (pce), etc. When compared to traditional materials and various other methods the pce of DSSCs is average. For instance, solar cells made using the six‐junctions inverted metamorphic structure have reported pce up to 47.1% under special incident conditions.^[^ ^85^ ^]^ Whereas the production scale multicrystalline or polyscrystalline Si solar cells exhibit pce between 9–15%.^[^ ^86^ ^]^ Photoconversion efficiencies at higher levels always have a cost factor attached to them due to the integration of exotic materials. Thereby lowering their realization of economies of scale. Here, DSSCs overcome the average power efficiencies due to their lower cost, flexibility, simple fabrication, and lightweight.^[^ ^83^, ^87^, ^88^ ^]^ The utilization of nanofibers as a buffer layer is another aspect of improvement in increasing the efficiency of organic solar cells to improve the charge transfer character of the solar cells. Mohammad Ali Haghighat Bayan et al. have followed this strategy by using a simple and effective two‐step process to sandwich hollow polyacrylonitrile (PAN) nanofibers to improve power conversion efficiency in high‐efficiency bulk heterojunction (BHJ) solar cells.^[^ ^89^ ^]^ This particular improvement in design based on nanofibers showed a 40.3% improvement in open‐circuit voltage and a 48.5% improvement in power conversion efficiency.^[^ ^89^ ^]^

**Figure 4:**
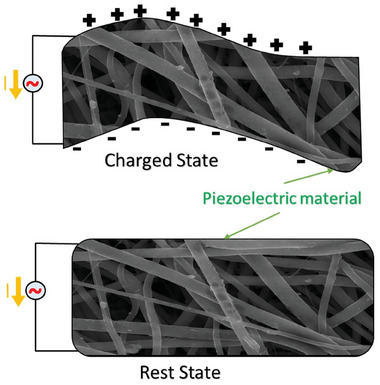
Operational mechanism of piezoelectric materials where bending/stretching the materials leads to a cycle between various phases for instance in the case of polyvinylidene fluoride (PVDF), a typical polymer used to make piezoelectric active nanofibers.

Another energy production area where nanofibers have taken the stage are piezoelectric and triboelectric nanogenerators, especially for flexible wearable energy harvesting devices which are easily integrated into existing structures to scavenge energy from motion and pressure changes (**Figure** **4**). Common piezoelectric materials that can be found as ceramics and polymers can undergo polarization when subjected to external mechanical stress leading to the generation of electric potential or minute displacements at a macroscopic level under an electric field. As seen in the drawn scheme (Figure 4) application of bending stress on the commonly piezoelectric polymer polyvinylidene fluoride (PVDF) creates potential difference across opposite surfaces, along and against the direction of applied force.^[^ ^90^ ^]^ When a load is connected across, this potential difference can be used to drive electrons and generate electricity for a wide range of small electronic devices.

Piezoelectric nanofibers have been developed as solutions to overcome the drawbacks of piezoelectric ceramic materials such as mechanical stability, restricted compositions, nonconformity, scaling up, and biocompatibility issues.^[^ ^91^ ^]^ Polymers such as polyvinylidene fluoride (PVDF), and styrene–ethylene–butylene–styrene (SEBS) have been excellent replacements offering comparable piezoelectric properties. Using these polymers alone or in combination with inorganic functional agents, researchers have been able to extract usable energy for powering miniaturized devices. Jiseul Park and the group developed a flexible poly(vinylidene fluoride‐co‐trifluoroethylene) (P(VDF‐TrFE) copolymer nanofibers‐based piezoelectric energy harvesting device. They leveraged the standard properties of nanofibers along with morphotropic phase boundary (MPB) characteristics of P(VDF‐TrFE) of various copolymer compositions to obtain a piezoelectric device capable of producing max voltage ranging from 20 V with a current output of 210 nA.^[^ ^92^ ^]^ When P(VDF‐TrFE) copolymer nanofibers were modified by the addition of piezoelectric nanocrystals such as inorganic perovskite halide CsPbBr_3_ and carbon nanotubes (CNTs) their performance was improved, which was confirmed via analysis under cyclic load. The performance of the fibers when compared to the fibers without the addition of the fillers showed a peak output voltage that was 17.6% better while the current output was 10.5 times increased.^[^ ^93^ ^]^ Perovskite/poly(vinylidene fluoride‐co‐hexafluoropropylene) (PVDF‐HFP) and SEBS nanofiber electrospun composite possess excellent electrical and mechanical properties to be used as piezoelectric energy generators. In this study, smart nanofibers were fabricated using Cs_3_Bi_2_Br_9_ perovskites as electron acceptors and local nucleating agents for the crystallization of polymer, which in turn enhances the electron trapping capacity and polar crystalline phase in nanofiber composite. With an output of 400 V, 1.63 µA cm^−2^, and 2.34 Wm^−2^ the device produced ample performance and showed comparable mechanical and surface properties for applications in biomechanics‐powered devices, wearables, and smart textiles.^[^ ^94^ ^]^ Using a combination of ZnSnO_3_ decorated CNTs immobilized on P(VDF‐TrFE) nanofibers Kang and group, leveraged the techniques of surface modification and electrospinning to design smart energy harvesting and sensing devices. They fabricated a device with simple assembly by sandwiching the acceptor and electrode layers and vacuum sealing inside a packaging film. The device was flexible and could be fixated on various surfaces including human skin without interfering with the nanofibers mat. Using normal body movement and bending testing, the authors were able to generate a stable output. Once the layers on the functionalized nanofibers mat were tripled, the generated energy output showed a significant increase in the voltage to 97.5 V and current at 1.16 µA. Interestingly, they managed to facilitate a pulsed detection of minute displacement using the device. Therefore, displaying the capability of such devices for sensing and harvesting energy sustainably and effectively.^[^ ^95^ ^]^

**Figure 5:**
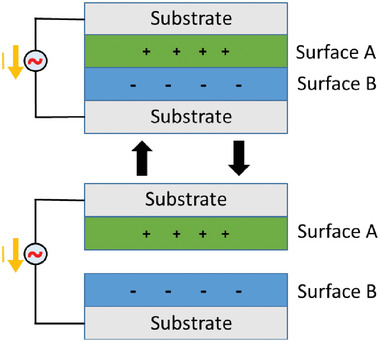
Typical arrangement of layers and charging mode for triboelectric nanogenerators (TENG). The vertical movement separates the layer creating a potential between oppositely charged surfaces, allowing the transfer of electrons.

**Figure 6:**
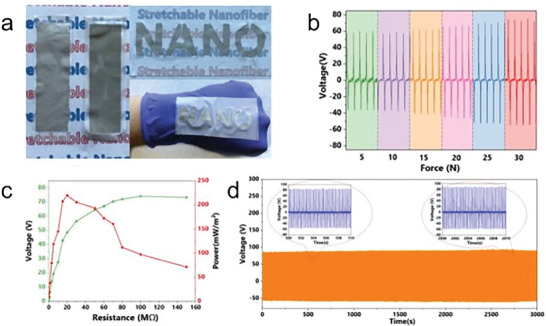
Properties and performance of stretchable nanofiber based TENG including a demonstration of the flexibility of the conductor a), the performance under different applied forces b), the dependency of voltage and power from resistance c) and the output durability under continuous measurements d). Reproduced (Adapted) with permission.^[^ ^97^, ^98^ ^]^ Copyright 2023, Elsevier.

Triboelectric energy generation is another sustainable method where nanofibers have increasingly been proven an effective solution (**Figure** **5**). Triboelectric energy nanogeneration has been described as a contact electrification phenomenon where moment contact between surfaces of a selection of materials can lead to the accumulation of polarizing charges, such as Surface A and Surface B in Figure 5. These charges create a potential difference across the load, which is generally connected on the surface opposite to the contact surface to minimize the charge loss and hence decrease in potential difference. Through various studies, a comprehensive list of materials that offer good triboelectric nanogenerators (TENG) performance is already available to design TENG devices.^[^ ^40^ ^]^ The dielectric nature of the polymer used for the fabrication of nanofibers is excellent for triboelectric applications. Here, PVDF polymer‐based nanofibers have emerged as suitable candidates for incorporation in triboelectric devices. As opposed to piezoelectric surfaces, triboelectric surfaces require the presence of two distinct surfaces one of which acts as a donor and the other as an acceptor. The use of PVDF polymer nanofibers along with nylon‐based films has been shown to demonstrate viable triboelectric performance. The Shi and group in their recent study showed that the incorporation of graphene in the PVDF nanofibers matrix offers a peak output voltage of ≈1511 V and a maximum peak power density of ≈130.2 Wm^−2^. The authors assembled a simple spring‐loaded device with multiple triboelectric layers and electrodes, which demonstrated eight times better performance than the TENG device in the absence of graphene.^[^ ^96^ ^]^ The versatility of electrospinning allows the deposition of nanofibers on a range of materials and morphology. Exploiting this advantage of fabrication Lee et al. have developed a highly flexible TENG device through the direct deposition of PVDF‐TrFE nanofibers on the multi‐walled carbon nanotubes (MWCNTs)/poly(dimethylsiloxane) (PDMS)/silver nanowires (AgNWs) composite electrodes. This mitigated the need to perform post‐processing or assembly of the structure in the form of TENG layers. The PDMS layer provided the adhesive force to keep the composite stable and decrease Young's modulus, hence increasing the flexibility of the devices. The output of the device with a cross sectional area of 30*30 m^2^ was measured at 5.28 Wm^−2^, which is relatively less than the other examples. However, their integrated approach promises a smoother fabrication process. Ideally, allowing the direct printing of TENG layers on the existing substrate surfaces, with little modifications.^[^ ^97^ ^]^ Due to the comparability and similarity in materials’ requirements of piezoelectric and triboelectric principles, it can often be found that the same materials or their combinations have been proven to be successful for utilization in both types of energy harvesting devices. Li et al. show an example of this familiarity by employing PVDF‐HFP electrospinning and electrospraying of SEBS for the fabrication of the TENG device, a composition utilized by Lee et al.^[^ ^97^ ^]^ By electrospraying SEBS on the surface of the fibers, the authors in the study were able to increase the stretchability and hydrophobicity of the PVDF‐HFP fibers networks. The layer was connected to the printable electrode consisting of gallium indium, tin particles, and silver flakes. Subsequently, the TENG device showed a good output of 85 V and a power density of 219.66 mW m^−2[^ ^98^ ^]^ (**Figure** **6**). TENG devices similar to their piezoelectric counterpart suffer from low energy efficiency, which renders them unusable for commercial applications. There have been many methods proposed to overcome this challenge. One of the ways is to introduce triboelectric layers with opposite polarities possessing a large surface area and high dielectric coefficient. By introducing cetrimonium bromide (CTAB) and dodecyl trimethyl ammonium bromide (DTAB) as doping agents to PVP nanofibers providing a positive dielectric layer, and combining it with a negative dielectric layer of PAN nanofibers, the study performed by Ozen et al. showed a significant increase in energy harvesting potential of TENG device. When 5% CTAB and DTAB were added to PVP nanofibers, it showed the best output measured at 500 and 515 V respectively. Therefore, the introduction of surfactants in the matrix of the triboelectric nanofibers layer can be effective in bringing the output to usable levels.^[^ ^99^ ^]^ Recently, Timusk et al. showed a two‐step synthesis method to prepare a polymer film from glycerol and sebacic acid precursors in a 1:1 molar ratio, which offers a material possessing excellent triboelectric properties besides mechanical and biological properties suitable for use in a wide range of applications.^[^ ^100^ ^]^ Therefore, providing a new avenue to bring advancement to TENG expertise.

Nanofibers have a high potential for improving energy storage solutions. The shortcomings faced by energy storage devices such as batteries and supercapacitors such as limited transfer and diffusion of ions due to low surface area, low electrolyte uptake capabilities, and the thermal shrinkage of the separators can be addressed up to a certain extent by the applications of hybrid nanofibers.^[^ ^101^, ^102^, ^103^, ^104^ ^]^ Recently, carbon nanofibers or carbon nanotubes modified nanofibers are used often to overcome challenges of energy storage. Raza et al. through their effort have successfully used the coaxial electrospinning method to synthesize copolymer poly(acrylonitrile‐*co*‐*β*‐methylhydrogen itaconate) and use it simultaneously as a precursor for preparing hollow flexible carbon nanofibers. Additionally, using the in situ reduction of MnO_2_, they were able to immobilize it throughout the fibers, which in turn were used as electrodes for solid‐state supercapacitors. The supercapacitor exhibited a maximum specific energy of 59.15 Wh kg^−1^ at a specific power of 1575 W kg^−1^.^[^ ^105^ ^]^ Post‐processing of nanofibers to prepare hierarchical porous carbon nanofibers has been shown to improve the electrochemical properties due to synergy on inherent nanofiber's surface area and pore volume. Here, Hu and group using simple blend electrospinning obtained MgCO_3_/Ni(CH_3_CO_2_)_2_/PAN nanofibers. These nanofibers were annealed and carbonized under a nitrogen atmosphere, which resulted in the carbonization of PAN additionally, the reduction of Mg and Ni.^[^ ^106^ ^]^ Further treatment of fibers initially, with base and later with acid activated the inorganic functionality and removed the excess Ni to provide a hierarchal porous structure. By doing the standard supercapacitor measurements the authors established that the samples delivered a specific capacitance of 287 F g^−1^ at 0.5 A g^−1^, a rate capacitance of 196 Fg^−1^ at 100 Ag^−1^ and improved capacity retention of 95.4% at 5 A g^−1^ after 10000 cycles. Furthermore, the fibers were used as anode for lithium‐ion batteries where they displayed a reversible capacity of 1495 mAh g^−1^ at 0.1 A g^−1^, superior cycle stability, and a capacity of 391 mAhg^−1^ at 10 Ag^−1^ for 1100 cycles. These results demonstrate the dual purpose of these fibers for both the supercapacitor and lithium‐ion battery applications.^[^ ^106^ ^]^ Similarly, thermal post‐processing of NiMoO_4_‐encapsulated carbon nanofiber performed by Amiri et al. after electrospinning resulted in hybrid nanomaterial effective for use in supercapacitors. The thermally treated nanofibers were used as electrodes in symmetric supercapacitors and their performance was analyzed. With this assembly, the authors obtained a specific capacity of 122.5 F g^−1^ at 1 Ag^−1^ and an energy density of 43.9 Wh kg^−1^ at a power density of 1567.9 W kg^−1^.^[^ ^107^ ^]^ Another field that has been improved with the implementation of nanofibers is the separator modules used in lithium‐ion batteries. Specifically, the introduction of lithium‐sulfur batteries, which overcome some drawbacks of lithium‐ion batteries but come with their issues requiring support from advanced materials to expand their adoption. Zhou et al. addressed one of the issues using polyvinyl alcohol/poly(lithium acrylate) (C‐PVA/PAA‐Li) composite nanofibers as separators in rechargeable lithium‐sulfur (Li—S) batteries. Through the combination of electrospinning, thermal cross‐linking, and in situ lithiation C‐PVA/PAA‐Li composite nanofiber membrane was fabricated possessing highly porous structures and offering high ionic conductivity.^[^ ^101^ ^]^ Moreover, the charge transfer resistance was minimized along with the growth of lithium dendrites, which can impair the battery recharging capacity. The separator showed improved ionic conductivity and Li^+^ diffusion coefficient, overall leading to a reduction in decay rate and an increase in cyclic performance.^[^ ^101^ ^]^

Nanofibers electrospun from a combination of poly‐D,L‐lactide (PDLLA), and cellulose nanocrystals are another versatile example of sustainable materials used in the improvement of lithium‐ion battery technology. Laezza and the group have shown through their recent study that simple blend electrospinning of nanofibers with appropriate materials can lead to an improvement in electrolyte uptake, thermal stability, and electrochemical stability. By using cellulose‐immobilized nanofibers as separators, they displayed the ecological and environmental viability of electrospun nanofibers.^[^ ^108^ ^]^

Through the enhanced electrocatalytic properties provided by hybrid nanofibers, there has been a positive impact on hydrogen fuel cell technology. Owing to the functionalization of PAA nanofibers using a coating sulfonated tetrafluoroethylene‐based fluoropolymer‐copolymer (Nafion) and platinum ink, the oxygen reduction reaction (ORR) kinetics and high current density H_2_ performance was improved in fuel cell electrochemical tests.^[^ ^109^ ^]^ One of the primary challenges in hydrogen fuel cell technology is the high hydrogen crossover, which can react with oxygen to degrade Nafion, frequently used as a membrane. By combining the electrospun fibers made from phosphonated polypentafluorostyrene (PWN70) and reinforcing Nafion, it has been shown that they performed better in fuel cell analyses. The H_2_ crossover was reduced at atmospheric pressure without sacrificing the proton conductivity.^[^ ^110^ ^]^ Furthermore, the thermal processing of nanofibers produced through facile blend electrospinning of N, P dual‐doped molybdenum carbide and PVP resulting in porous metal‐doped carbon fibers have shown an improved catalytic activity and stability in the medium used for hydrogen evolution reaction.^[^ ^111^ ^]^

Overall, electrospun nanofibers offer a versatile platform for energy harvesting, conversion, and storage applications. Offering new material combinations with scale‐up potential possessing superior properties compared to traditional materials allowing for higher efficiency in energy conversion processes.

### 3.3 Catalysis and Environmental Remediation Application

The continuously increasing environmental pollution leads to the need for efficient materials for environmental remediation. These novel materials should combine cost and energy efficiency with recyclability and in the ideal case a sustainable production method. Electrospun smart hybrid nanofibers are promising materials to fulfill these requirements.^[^ ^112^, ^113^, ^114^, ^115^, ^116^ ^]^

**Figure 7:**
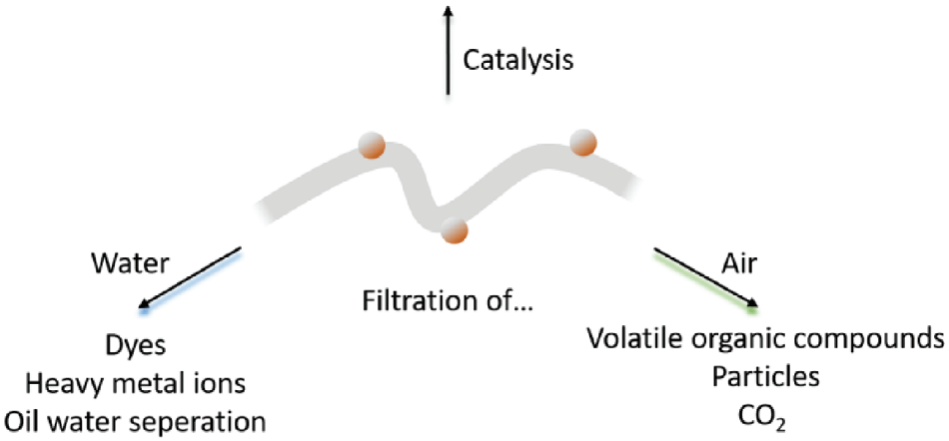
Catalysis and environmental remediation applications of hybrid nanofibers.

For environmental remediation, the pollutants can be removed from the air or water simply via filtration or they can be converted directly by catalytic functionalities incorporated in the hybrid nanofibers (**Figure** **7**). Electrospun nanofibers can be combined with metal oxide nanoparticles like TiO_2_, carbon nanotubes, precious metals, metal‐organic frameworks (MOFs), or biological agents.^[^ ^113^ ^]^ MOFs are already known to be promising catalysts and their incorporation in fibers leads to easier recyclability and increased adsorption of pollutants.^[^ ^117^, ^118^, ^119^ ^]^ For enhanced mechanical stability or other specific requirements, additional components like carbon nanotubes can be incorporated.^[^ ^120^ ^]^

Some examples of catalytically active hybrid materials are TiO_2_‐ acetylacetonate and silica nanoparticles incorporated in PVP nanofibers via electrospinning. For instance, these hybrid systems can be used for oxidative degradation of methylene blue that has shown activity even in the dark.^[^ ^121^ ^]^ Additionally, photocatalytically active ZnO nanoparticles have been incorporated in cellulose nanofibers and displayed promising catalytic activity when tested with rhodamine.^[^ ^122^ ^]^ Another example is the incorporation of gold nanoparticles in polypropylene nanofibers modified with polyethylene glycol for the reduction of methylene blue in the presence of sodium borohydrate.^[^ ^123^ ^]^ Polyacrylonitrile nanofibers with incorporated 2‐Methylimidazole were used for immobilization of Zeolite imidazolate framework‐8 (ZIF‐8) where catalytically active silver nanoparticles were grown. These hybrid fibers showed good repeatability toward the p‐nitrophenol conversion.^[^ ^124^ ^]^ Recently, hybrid nanofibers for enhanced hydrogen evolution reactions were introduced. Through the carbonization of electrospun PAN nanofibers immobilized with Ni and Pt ions, the ions were reduced to nanoparticles, which led to a highly effective electrocatalyst for water splitting.^[^ ^125^ ^]^ Palladium embedded nitrogen doped carbon nanofibers were prepared by electrospinning of PVP with palladium cations and polyimide followed by carbonization to produce effective catalysts for the Suzuki reaction. The hybrid nanofibers showed excellent yields and could be recovered by filtration and reused 5 times.^[^ ^126^ ^]^

Dyes are complex organic molecules found commonly as pollutants that need an efficient cleaning process, which is missing due to their complex structures and low biodegradation. They hinder photosynthesis when absorbed by plants due to the lowering of light penetration and bioaccumulation.^[^ ^117^ ^]^ It has been shown that through the immobilization of ZIF‐8 on chitosan/polyvinyl alcohol nanofibers malachite green could be removed from wastewater.^[^ ^127^ ^]^ The presence of UiO‐66 within organic nanodiamonds in chitosan/polyvinyl alcohol nanofibers showed the ability to remove cationic methylene blue as well as anionic Congo red dyes in 6 adsorption and desorption cycles.^[^ ^128^ ^]^ The zeolitic imidazole framework‐67 (ZIF‐67) within electrospun PAN fibers was shown to adsorb dyes such as malachite green, which is used in textile, leather, and paper production. Additionally, the membranes were tested with Congo Red as well as Basic Fuschin with adsorption capacities of 1305, 849, and 730 mg g^−1^. The membranes could be regenerated four times by simple washing with ethanol and still showed 92% of the original adsorption capacity, which improves the practical application in industry in comparison to MOF powders.^[^ ^129^ ^]^ The regeneration of nanofibrous membranes was also shown for a system where anionic bio‐MOF‐1 was incorporated in PAN nanofibers. This hybrid system was developed for the removal of cationic dyes such as methylene blue from wastewater and could be regenerated by ion exchange processes up to five times. The adsorption of only cationic dyes was explained by the free NH_2_ (CH_3_)_2_^+^ channels of the MOF in combination with the nucleophilic PAN making it possible to separate such dyes from mixtures.^[^ ^130^ ^]^ Fibers made of polystyrene were decorated with beta‐cyclodextrin to capture organic molecules in solutions by inclusion complexation. Here, phenolphthalein was used as a model organic compound. A high dependency of the filtration efficiency regarding the distribution of the beta‐cyclodextrin was described. With higher contents of beta‐cyclodextrin at the surface a faster adsorption was observed with up to 65% decrease in the concentration of the phenolphthalein.^[^ ^131^ ^]^ Another example of a hybrid system for the absorbance of dyes from water is introduced by Scaffaro et al. who electrospun poly(vinylidene fluoride‐co‐hexafluoropropylene) with graphene oxide. CNT hybrid nanofibers were produced, which adsorbed methylene blue from water and enabled the possibility of analyzing the concentration of the dyes at the same time. The maximum adsorption capacities ranged from 120 to 555 mg g^−1^. In dependency of the amount of adsorbed dye the conductivity of the system increased linearly.^[^ ^132^ ^]^

**Figure 8:**
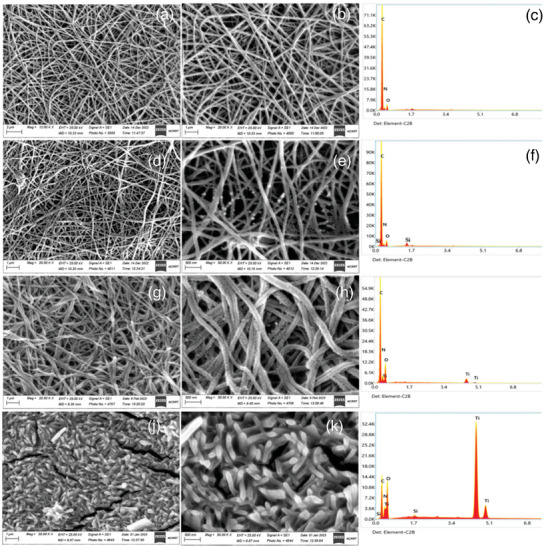
FE‐SEM images of polyamide nanofibers a,b), polyamide nanofibers with SiO_2_ nanoparticles d,e), polyamide nanofibers with TiO_2_ nanoparticles g,h), and polyamide nanofibers with SiO_2_ and TiO_2_ nanoparticles j,k) with associated EDX spectra c,f,i,l). Reproduced with permission.^[^ ^135^ ^]^ Copyright 2024, MDPI.

Su et al. introduced PAN nanofibers blended with TiO_2_ nanoparticles that were able to adsorb organic molecules and degrade them at the same time by UV light irradiation. The photocatalytic degradation was shown for phenol and parameters influencing the degradation like pH and initial concentration of phenol were identified. Additionally, the fibers were stable and the authors were able to recycle the active agents showing a sustainable and environment friendly approach.^[^ ^133^ ^]^ Degradation of pollutants in water was also shown with nanofibers containing titanium dioxide, silver, and boron nanosheets as well as halloysite nanotubes. The system showed the possibility of breaking down non‐degradable compounds such as acetaminophen in wastewater via oxidation processes. The photocatalytic degradation showed an efficiency of 99.29% after irradiation for 2 h.^[^ ^134^ ^]^ Saleh et al. recently introduced another multifunctional electrospun filtration membrane, which did not degrade the pollutants but added antibacterial properties. They used polyamide nanofibers loaded with silica, titanium dioxide, or a mixture of both. The adsorption was tested with methylene blue and was up to 27 mg g^−1^. The antibacterial properties were tested with different bacteria populations like *Escherichia coli* or *Klebsiella pneumonia* (**Figure** **8**).^[^ ^135^ ^]^

The removal of pollutants such as heavy metal ions from water by nanofibers membranes takes place through multiple approaches such as employing appropriate pore sizes, favored adsorption of pollutant particles or leveraging the interaction of pollutants with molecules added to the membranes.^[^ ^117^ ^]^ Jamshidifard et al. showed the removal of heavy metal ions by using UiO‐66‐NH_2_ MOF incorporated in PAN /chitosan nanofibers. The UiO‐66‐NH_2_ MOF were synthesized in microwave and added to the polymer solution before electrospinning. The filtration was investigated for different parameters such as MOF content, pH value, temperature and initial concentration of Pb(II), Cd(II) and Cr(IV) ions with adsorptions up to 441.2 mg g^−1^, 415.6 and 372.6 mg g^−1^, respectively.^[^ ^136^ ^]^ Cadmium adsorption was also achieved in PVP nanofibers decorated with TiO_2_/ZrO_2_. The fibers showed a high surface are of 248 m^2^ g^−1^ and showed higher adsorption capacities as well as lowered pH sensitivity after surface functionalization via phosphonic acid coupling reactions.^[^ ^137^ ^]^ Nano‐fibrous metal‐organic frameworks, MOF‐808 incorporated in PAN nanofibers showed the ability to adsorb heavy metal ions as well. Here, different affinities depending on the metals were shown. Additionally, an influence of absorbance efficiency by coexisting cations was observed illustrating the complexity of wastewater treatment.^[^ ^138^ ^]^ Through incorporation of MWCNTs for enhanced stability and with zero valent iron nanoparticles as the active agent, a hybrid system composed of polyacrylic acid/polyvinyl alcohol fibers has been shown to be effective for the removal of heavy metal ions such as copper ions from water.^[^ ^139^ ^]^ Razzaz et al. introduced Chitosan/TiO_2_ fibers for the removal of Pb‐ and Cu‐ions. The adsorption capacities were up to 579.10 and 715.70 mg g^−1^ respectively. The authors describe differences in the adsorption capacities regarding the preparation methods. They compared fibers with embedded TiO_2_ nanoparticles, showing the better adsorption capacities and fibers which were coated with TiO_2_ nanoparticles by dipping the fibers in the dispersion.^[^ ^140^ ^]^ Thiol‐functionalized mesoporous polyvinyl alcohol/SiO_2_ fibers were used for heavy metal ions removal as well. The adsorption capacity for Cu(II) ions was up to 489.12 mg g^−1^ and the fibers showed recyclability in 6 cycles by stirring in HCl and washing.^[^ ^141^ ^]^ Recently biomembranes consisting of Poly(butylene succinate) and poly(lactic acid) with added Cloisite® nanoclays, which are organophilic phyllosilicates were tested for removal of toxic metal ions like Cd(II), Zn(II), Cu(II), Ni(II), and Mn(II) from water. Here a selectivity series could be observed, with efficiencies between 38% and 6% after thermal modification.^[^ ^142^ ^]^ Copper ion uptake was also shown with biodegradable films of zein and polyvinyl alcohol loaded with nanohydroxyapatite. The incorporated hydroxyapatite nanoparticles improved the hydrophobicity and thermal stability of the membranes.^[^ ^143^ ^]^ PAN fibers loaded with ZIF‐94 were tested for the adsorption of cobalt ions and showed good recyclability in four cycles with an adsorption efficiency of 95% of the initial uptake. Regeneration of the mats was done with nitric acid as desorbent.^[^ ^144^ ^]^ Arsenic contaminations could effectively be lowered by polysulfone membranes with graphene oxide particles. These hybrid systems showed improved hydrophilicity and increased adsorption rates of arsen compared to the pure fibers. The influence of pH on the adsorption was studied and showed improved adsorption of arsen at higher pH values.^[^ ^145^ ^]^ Amidoximed polyacrylonitrile/ZIF‐67 incorporated in PAN was investigated for capturing uranium ions in the sea‐ and wastewater. At pH 4, the adsorption was up to 498.4 mg g^−1^ in aqueous solutions and 2.03 mg g^−1^ in natural seawater.^[^ ^146^ ^]^

**Figure 9:**
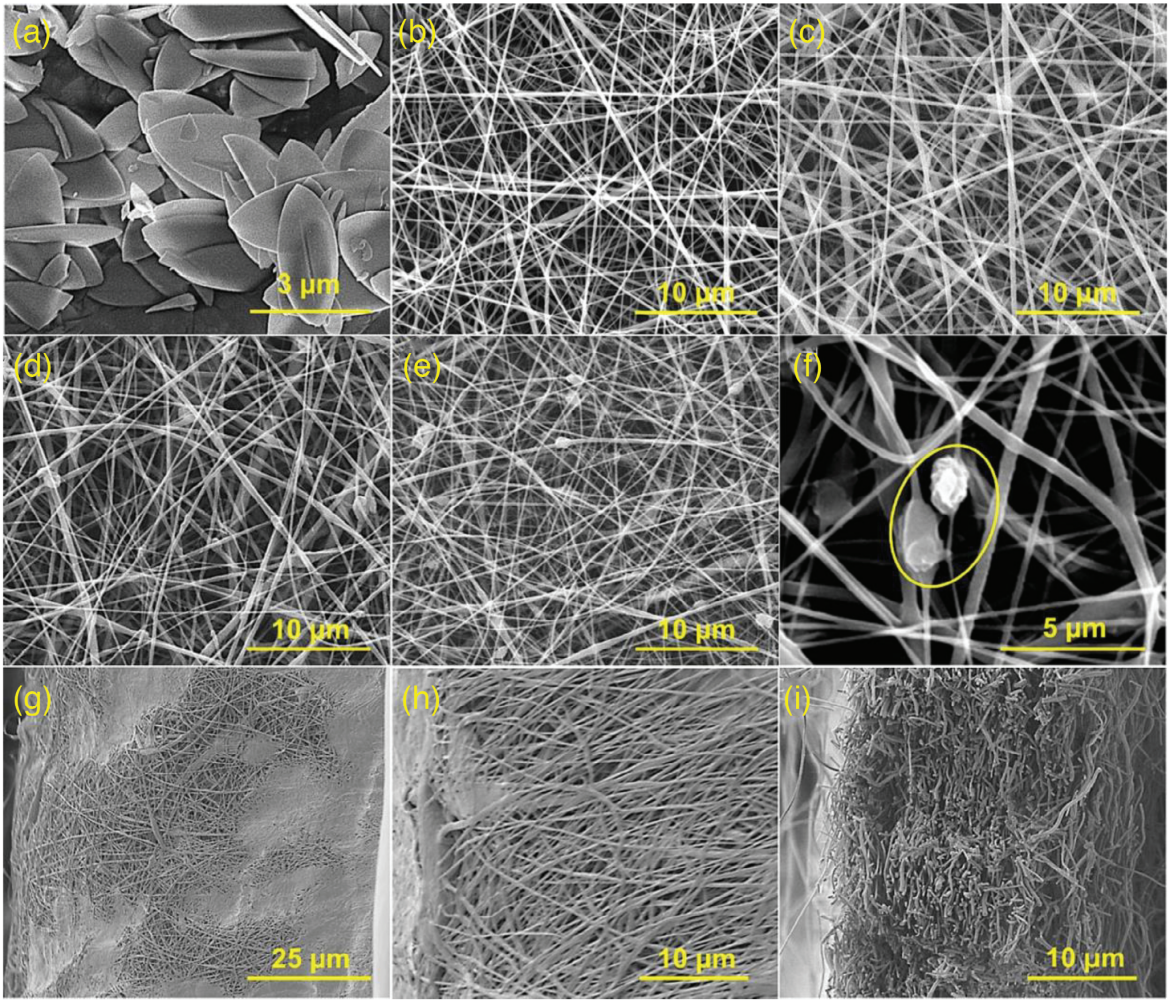
SEM images of the zeolitic imidazolate framework crystals a), the poly(vinylidene fluoride‐cohexafluoropropylene) nanofibers b), and their composite membranes with 1 wt.% ZIF c), 3 wt.% ZIF d) and 5 wt.% ZIF added e,f). Cross sectional SEM images of pristine fibers g), fibers with 1 wt.% ZIF h), and with 5 wt.% ZIF i). Reproduced with permission.^[^ ^155^ ^]^ Copyright 2024, Elsevier.

Along with water pollution, air pollution is another cause of major health issues around the globe, highlighting the importance of filtration of organic compounds or particulate matter. The advantages of electrospun membranes like the porous structure and high filtration efficiency might be promising but the usage of organic solvents which might be toxic or hard to recycle is still a challenge.^[^ ^114^ ^]^ Overcoming this issue the usage of electrospun hybrid nanofiber mats as promising class of materials for air filtration in facemasks was described. The porosity as well as the functional agents can be adjusted to reach certain levels of regulatory standards and adjust the filtration mechanisms.^[^ ^147^, ^148^ ^]^ For capturing volatile organic compounds from air (VOCs) polybenzimidazole fibers with different sizes of platinum particles can be used. Polybenzimidazole is thermally stable and shows a high porosity as an electrospun membrane. The platinum nanoparticles achieved conversion ≈90% at temperatures below 335 °C using model VOCs like ethanol, acetone and toluene.^[^ ^149^ ^]^ Yu et al. introduced PAN fibers combined with SiO_2_ aerogel with high adsorption of VOCs. The surface area was improved by the incorporation of the SiO_2_ aerogel with honeycomb like structure, promoting the adsorption capability for the tested VOCs, namely chloroform, xylene, formic acid and methanol. The membranes were recycled ten times by degassing at 35 °C under ambient pressure.^[^ ^150^ ^]^ Another system for removing VOCs is a fly ash/polyurethane hybrid material where the recyclability by adsorption and desorption cycles was shown. The fly ash is a byproduct of thermal plants and was easily incorporated by blend solution electrospinning. With increasing amount of fly ash the adsorption capacity increased up to 2.79 times compared to the pristine fibers.^[^ ^151^ ^]^ Moreover, pollutants in the air comprise of fine particles such as silicates, sulfates or carbon compounds. Fine particle filters can be produced by incorporating titania nanoparticles in electrospun polysulfone. This system shows a filtration efficiency of 99997%. The filtration efficiency as well as the pressure drop could be fine‐tuned by variations of the surface composition and the hierarchical structure. These membranes showed superhydrophobic properties and formation of mesopores by addition of the nanoparticles.^[^ ^152^ ^]^ Hybrid materials of poly(vinyl alcohol)/poly(acrylic acid) fibers with silica and silver nanoparticles have shown promise as particle filters combined with antibacterial and antiviral activities. These membranes were produced by environmentally friendly green electrospinning with post modifications like thermal treatment for cross‐linking and UV irradiation for the reduction of silver ions.^[^ ^153^ ^]^ Huang et al. recently introduced poly(L‐lactic acid) fibers with copper nanoparticles, synthesized in an environmentally friendly process. These hierarchically porous membranes were tested for antibacterial applications as air‐permeable and superhydrophobic bendable material to restrict the spread of diseases. The membranes were synthesized by electrospinning of the polymer followed by a coating step to introduce the copper nanoparticles. A post modification was done by incubation of the membranes either in L‐ascorbic acid or in copper acetate, resulting in antibacterial effectiveness's of 99.9% and 90% for Staphylococcus aureus and 99.999% and 99.9% for Escherichia coli, respectively.^[^ ^154^ ^]^ Additionally, some hybrid nanofiber systems for CO_2_ adsorption and catalysis were introduced recently. Kim et al. described nanofibrous membranes of poly(vinylidene fluoride‐co‐hexafluoropropylene) fibers including 2D ZIFs (**Figure** **9**). By the combination of the CO_2_ adsorption capacity of the ZIF with the porosity of the polymer fibers, an energy‐efficient CO_2_ separation can be enabled. The membrane was produced by blend electrospinning with ZIF loadings between 0.5 and 5 wt.%.^[^ ^155^ ^]^ The electroreduction of CO_2_ to formate was shown with BiCu/carbon hybrid nanofibers. This membrane was produced by blend electrospinning followed by calcination and showed high activity and selectivity with Faradiac efficiencies up to 88.10%.^[^ ^156^ ^]^ The photocatalytic reduction of CO_2_ was shown with core‐shell heterojunction g‐C_3_N_4_/NaNbO_3_ fibers by Wang et al. NaNbO_3_‐ PVP nanofibers were modified with urea and calcinated to obtain fibers with improved photocatalytic activity for the reduction of CO_2_ to CH_3_OH. NaNbO_3_ acts as photocatalyst but its application is limited so far caused by a fast recombination and low sensitivity to visible light. The combination with g‐C_3_N_4_ leads improved photocatalytic properties with methanol yields of 12.86 µmol gh^−1^.^[^ ^157^ ^]^

The separation of oil from water is another application in the field of environmental remediation where nanofibers have been quite effective. PAN nanofibers with nanocrystalline zeolite imidazole frameworks that exhibit switchable wettability depending on the pretreatment have been fabricated to separate water‐oil emulsions.^[^ ^158^ ^]^ Furthermore, a superhydrophilic membrane has been produced by incorporation of MOF‐5 in coal‐based fibers and was shown to separate oil‐water mixtures beside adsorption of organic dyes.^[^ ^159^ ^]^ Liang et al. introduced a nanofiber membrane which combined superhydrophilic and self‐cleaning properties. The membrane was produced by electrospinning of PAN with different amounts of Co_3_O_4_, ranging from 0 to 10 wt%. A separation efficiency of 99% was shown for surfactant‐stabilized emulsions. An integrated catalytic degradation was shown for methylene blue by addition of peroxymonosulfate, which additionally activated the self‐cleaning properties.^[^ ^160^ ^]^

Electrospun hybrid nanofibers show great potential for environmental remediation offering a huge variety of implementable inorganic additives as active agents. However, a detailed understanding of the mechanisms as well as improved material designs are required for optimizing performance and increasing effectivity. This will lead to the realization of multifunctional hybrid materials possessing the ability for adsorption and partly an integrated conversion of pollutants by recyclable materials.

### 3.4 Controlled Drug Delivery and Tissue Engineering Application

**Figure 10:**
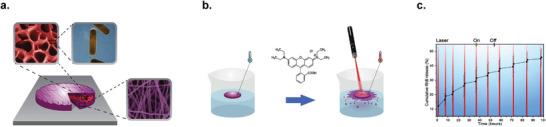
a) Scheme presenting the hybrid hierarchical nanoplatform: It involves a P(NIPAAm‐co‐PNIPMAAm) hydrogel that encompasses gold nanorods (AuNRs), which is then sandwiched between two nanofibrous PLA layers containing RhB. b) A schematic illustrating the temperature variation induced by laser irradiation, resulting in an increase in the release of RhB. c) The cumulative release of RhB following 10 cycles of laser irradiation is depicted in the graph. The vertical red lines indicate the 1 h duration of each laser irradiation cycle. Reproduced (Adapted) with permission.^[^ ^174^ ^]^ Copyright 2020, ACS Publications.

Conventional drug delivery platforms encounter critical issues, such as inconsistent release of drug molecules, suboptimal drug delivery, limited drug encapsulation, and reduced drug stability and bioavailability.^[^ ^161^, ^162^, ^163^, ^164^ ^]^ While nanofibrous membranes can alleviate these issues due to their unique properties (high aspect ratio, large surface area, tunable pore structures, high porosity, high drug loading capacity), nanocarriers that can actively release drugs only in response to specific stimuli are considered the ideal drug delivery systems. Compared to other nanocarriers like micelles, liposomes, and nanoparticles, electrospun fibers may not be suitable for bloodstream administration, but they can be customized for various delivery methods. These methods include oral, topical, transdermal, buccal, injectable, and implantable routes, allowing for both systemic and local effects.^[^ ^165^, ^166^ ^]^ The latest advancements in the field of organic‐inorganic hybrid nanomaterials have led to the development of electrospun nonwoven fibers that can deliver precise dosages of drugs to targeted areas through stimuli‐mediated mechanisms, enabling both spatial and temporal drug delivery. This type of on‐demand drug delivery system is commonly known as a smart or intelligent one because of its physical or chemical properties, which include being responsive to various stimuli and sensitive to different environments.  Smart delivery systems can be produced using a polymer that possesses dynamic properties that can be altered by temperature, pH, or humidity.^[^ ^167^, ^168^, ^169^ ^]^ Alternatively, a non‐responsive polymer can be used, and supplementary molecules or nanoparticles can be added to create a nanofibrous membrane with specific responses, such as electrical, biological, or optical.^[^ ^17^, ^170^, ^171^, ^172^, ^173^ ^]^ There has been a great deal of research done on this approach, and it has yielded some very promising outcomes. As a result, a wide range of multifunctional materials have been synthesized, and they have shown a lot of potential. For instance, polymers possessing macromolecular chains that undergo coil‐to‐globule transition above a temperature defined as lower critical solution temperature (LCST) have been widely employed to generate thermo‐responsive nanofibrous membranes due to their ability to trigger drug release. The LCST‐based mechanism of these polymers is triggered by the environmental temperature, which causes the polymer chains to switch from a hydrated, expanded coil conformation to a collapsed globular conformation. This classical concept has proven to be highly effective in drug delivery for cancer therapy and wound healing applications and continues to be an area of active research. In a recent study, Nakielski et al. have developed a hybrid nanoplatform comprising a sandwich‐like medicated (model drug – Rhodamine B (RhB)) PLA nanofibrous outer layer and thermo‐responsive poly(N‐isopropylacrylamide‐co‐N‐isopropylmethacrylamide) (P(NIPAAm‐co‐NIPMAAm)) inner hydrogel containing gold nanorods (**Figure** **10**a).^[^ ^174^ ^]^ By utilizing plasmonic hydrogel‐light interaction, the biomimetic nanostructured pillow can contract mechanically when required (Figure 10b). Such an ingenious scenario increases the temperature by up to 44 °C and accelerates water expulsion from the inner structure, resulting in rapid release profiles of the model drug (Figure 10c).

Moreover, during the pursuit of an appropriate stimulus to activate drug release under physiological or treatment conditions, Pan et al. discovered that a non‐responsive polymer or polymer blend's glass transition temperature (T_g_) could serve as an ideal parameter to regulate drug release.^[^ ^175^ ^]^ The authors achieved a well‐tuned T_g_ for a controlled drug release from nanofibers at 37 °C by varying the blending ratio of two selected polymers. The finding suggests that T_g_ can be a valuable tool in designing drug delivery systems that can be triggered at specific temperatures.

Another well‐studied stimulus for creating hybrid and smart drug delivery systems is pH; because the human body's pH values can vary in parts of the digestive tract, different organs, tissues, and cellular compartments.^[^ ^172^, ^176^, ^178^ ^]^ In specific cases, internal stimulation, such as a low pH in tumor tissues, can be exploited for drug delivery to the exact site of the tumor, allowing for targeted treatment of the affected cells. This strategy helps to ensure that the anti‐cancer drug is released only in the required area, thereby minimizing the risk of unwanted side effects.

For instance, Zhao et al. have developed a revolutionary system that enables smart tumor‐triggered controlled drug release.^[^ ^179^ ^]^ The system uses an electrospun hybrid structure with inorganic cap CaCO_3_ that is highly sensitive and controls the opening of pore entrances of drug‐loaded mesoporous silica nanoparticles (MSNs) inside poly(l‐lactide) (PLLA) nanofibers. By using pH‐triggered inorganic “caps” with CO_3_^2−^ functional groups on MSNs, the system can react instantly in the acidic tumor microenvironment to produce CO_2_ gas that can speed up the penetration of water to the inner cores of the PLLA nanofibers, facilitating drug release. Sometimes, controlled drug delivery systems, which encapsulate a single drug, might fail to achieve the all‐right desired therapeutic efficiency due to the rapid development of drug resistance in microorganisms. Most recently, engineered pH‐responsive bi‐compartmented Janus nanofibers with a synergistic combination of therapeutic drugs have been made to address these issues.^[^ ^38^ ^]^ The ability of two mutually independent regions made from pH‐responsive polyelectrolytes to co‐load multiple drugs was advantageous in designing smart drug delivery systems with an on‐demand sequential release. It is well known that the pH sensitivity of polyelectrolytes, thanks to the presence of acidic or basic functionalities on their polymeric backbone, allows them to dissociate into highly charged polymeric molecules when exposed to ionizing solvents or water.^[^ ^180^ ^]^ These functionalities can either donate or accept protons.

By manipulating the formulation and chemical composition of the polymer matrix, along with the precursor of inorganic components, it becomes possible to tune the sensitivity to stimuli finely. Intelligent nanofibers made with a multi stimuli‐responsive design have the capability to react to two or more signals at once, expanding their versatility in drug delivery. These combined responses, such as pH and temperature,^[^ ^181^, ^182^ ^]^ pH and light,^[^ ^183^ ^]^ redox‐ and pH,^[^ ^184^ ^]^ light and magnetic field,^[^ ^185^ ^]^ pH, temperature, and electric field^[^ ^186^ ^]^ can happen simultaneously or in a particular sequence, allowing the nanofibers to autonomously identify the required therapy and administer the necessary active ingredients with extraordinary accuracy, resulting in targeted drug delivery.

On the other hand, locally administrable, stimuli‐responsive nanofibrous membranes are innovative materials that hold great prospects in the field of tissue regeneration.^[^ ^171^ ^]^ These advanced membranes can be stimulated externally to precisely deliver therapeutic agents to lesion sites in a controlled manner, thereby promoting cell attachment and proliferation, owing to their morphological characteristics that are similar to those of the extracellular matrix (ECM).^[^ ^187^, ^188^, ^189^ ^]^ Additionally, they also exhibit intelligent and adaptive responses to alterations in the internal microenvironment, making them an ideal candidate for synergistic therapeutic interventions that combine multiple strategies to enhance therapeutic efficacy.^[^ ^190^, ^191^ ^]^ In this regard, Sasikala and her team have proposed for the first time a cutting‐edge implantable multimodal therapeutic nanofibrous membrane embedded with superparamagnetic graphene oxide (SPGO) hybrid to significantly reduce local tumor recurrence by means of pH‐responsive doxorubicin release over multiple cell cycles, along with magnetic field induced long‐term hyperthermia. This multifaceted nanocarrier not only fights against cancer but also acts as a biodegradable scaffold for preadipocytes, helping to create normal adipose tissues for breast reconstruction. It is also equipped with noninvasive monitoring capabilities through magnetic resonance imaging, enhancing patient care effectiveness.^[^ ^192^ ^]^

Injectable nanofibrous hydrogels are generating significant interest in drug delivery and tissue engineering due to their ability to be delivered to the target sites through minimally invasive methods that escape surgical complexities.^[^ ^193^, ^194^, ^195^ ^]^ A recent study within this framework has evaluated the safety and feasibility of minimally invasive intradiscal delivery of bone marrow‐derived mesenchymal stromal cells (BM‐MSCs) through fibrous microscaffold injectable carriers for treating degenerated nucleus pulposus tissue in a large animal model.^[^ ^196^ ^]^ Moreover, Rybak and colleagues have engineered a self‐healing, antibacterial hydrogel using Pluronic F‐127 and sodium alginate (SA). This innovative hydrogel can be precisely delivered to targeted tissues through injection. By encapsulating drug‐loaded nanofibers and polydopamine particles, the hydrogel effectively addresses the challenges associated with bacterial infections in wound healing.^[^ ^197^ ^]^

Guided bone regeneration (GBR) is a well‐established therapeutic approach for repairing bone defects. To enhance its effectiveness, nanofibrous GBR membranes integrated with photothermal therapy have emerged as a promising option. Therefore, Ma et al. conducted a study focused on enhancing bone regeneration using a combination of electrospun hybrid PCL nanofiber membranes and near‐infrared (NIR)‐triggered photothermal therapy.^[^ ^198^ ^]^ To achieve this, they utilized nanosized molybdenum disulfide (MoS_2_) as both a biocompatible osteogenic enhancer and a NIR photothermal agent, which has remarkable physiochemical properties and excellent bioactivities. Results from a study conducted on bone mesenchymal stem cells (BMSCs) in vitro and on rat tibia bone defects in vivo have confirmed that the use of PCL/MoS_2_ nanofiber membranes can accelerate osteogenesis and bone healing, leading to the restoration of bone defects.^[^ ^198^ ^]^

Excessive levels of endogenous reactive oxygen species (ROS) are frequently employed as a stimulus in pathological microenvironments that are responsive to tissue therapy and regeneration. Coronary artery blockages can lead to a severe cardiac condition known as myocardial infarction (MI), resulting in significant damage.^[^ ^199^ ^]^ To facilitate the heart's recovery from such damage, tissue engineering techniques have been developed, including the utilization of functional patches made from biodegradable elastomeric polyurethane with thioketal (PUTK) linkages that are ROS‐responsive. According to the research findings, pristine PUTK and the glucocorticoid methylprednisolone (MP) drug‐loaded PUTK fibrous patches offer exceptional protection to the myocardium against oxidative injury, resulting in a significantly higher survival rate of cardiomyocytes when compared to the non ROS‐responsive polyurethane fibrous patch group and MI group after the infarction injury for 24 h. Furthermore, the implantation of PUTK/MP fibrous patches for a period of 28 days proved to be an effective means of improving the reconstruction of cardiac functions, which led to an increased ejection fraction, decreased infarction size, and enhanced the revascularization of the infarct myocardium.^[^ ^200^ ^]^ From the above viewpoint, smart and multifunctional membranes composed of electrospun nanofibers have been demonstrated to be good candidates for tissue engineering applications. However, these membranes often present challenges, such as compact 2D architecture and a densely packed fiber layer with superficial pores, which may impede cell infiltration, growth, and nutrient flow. To address the limitations of current tissue engineering scaffolds, researchers are exploring innovative fabrication strategies to develop an ideal scaffold that closely replicates a natural ECM's structure and spatial topography like a 3D nanofibrous environment.^[^ ^201^, ^202^, ^203^, ^204^, ^205^ ^]^ In recent years, electrospinning technology has undergone tremendous advancements, leading to the development of 3D nanofibrous scaffolds with macroporous structures. The methodologies employed in this context encompass liquid and template‐assisted collection, self‐assembly of nanofibers, and layer‐by‐layer deposition, predominantly relying on modifying the collector's geometry or integrating sacrificial components through direct 3D electrospinning.^[^ ^206^, ^207^, ^208^, ^209^ ^]^ Furthermore, foaming technology, which involves gas generation in an aqueous solution, is a widely utilized post‐processing technique for transforming 2D nanofibrous membranes into porous, thicker structured 3D scaffolds.^[^ ^210^, ^211^, ^212^ ^]^ While numerous studies have delineated the notable impacts of 3D scaffolds with fibrous networks on cell growth and behavior in vitro or in vivo models,^[^ ^213^, ^214^, ^215^, ^216^, ^217^ ^]^ there is a lack of research on integrating stimuli‐responsive hybrid biomaterials that contain organic and inorganic compounds into 3D electrospinning. This integrated fabrication will propose a potential strategy for designing and building the next generation of bioactive 3D smart nanofibrous scaffolds with controllable alterations in their properties in response to both external and internal stimuli.

## 4 Conclusion and Future Prospects

Electrospinning is a powerful tool in nanofabrication to generate smart hybrid nanofibers with unique collective properties. Electrospinning offers precise control over the fiber's diameter, morphologies, composition, structure, porosity, and functionality. Smart nanofibers fabricated using electrospinning have far‐outreaching potential to influence the field of energy harvesting, storage, and conversion along with environmental remediation, catalysis, smart textiles, and healthcare, significantly. The advantage of possessing multiple functionalities within smart nanofibers promises innovative solutions for challenges because of the changing climate and the constraints of developing a sustainable society. Smart hybrid nanofibers offer flexibility to conform to the body and its movements making it a favorable platform for flexible electronics, improving energy density and performance of energy storage modules. The capability of nanofiber‐containing devices to leverage piezo and triboelectric individually or simultaneously can be used for creating diverse sensors, and capacitors for both health and environmental monitoring. Through electrospinning, it is possible to design hierarchical structures that allow integrated stretchable substrates for providing efficient TENG and PENG devices. Fabrication of hybrid nanofibers specifically with the inclusion of functional agents like perovskites and catalysts can be utilized for highly efficient photovoltaic devices. Further improving the reaction kinetics in fuel cells by affording novel compositions to maximize energy density in energy storage modules besides offering durability flexibility and sustainability.

Furthermore, the application of electrospun hybrid nanofibers in smart textiles holds vast possible uses driven by advancements in textile engineering. It offers transformation in multiple industries from healthcare and sports to environmental monitoring. One of the key prospects focuses on the potential developments for monitoring vital signs such as heart rate, blood pressure, and glucose via embedded multisensory. These miniaturized devices are designed with the goal of early detection of health issues, which when combined with targeted therapy can improve patient compliance and safety. Moreover, promoting faster healing via controlled drug release of growth factors and antimicrobial agents in a simultaneous responsive manner. Likewise, environmental sensing platforms can respond to environmental changes, such as pollution levels, temperature, and humidity, making them useful in environmental monitoring by embedding them into sustainable structures in buildings, and pipelines, providing early warnings of potential failures. The use of innovative nanofibers in smart textiles is a wide but immature field that needs intensive research and continued development to address current challenges, which will be crucial in realizing their full potential in everyday applications to enhance human well‐being, safety, and overall quality of life.

Selective capturing and efficient catalysis are additional advantages of smart nanofibers. This combination has been proven an effective tool for the remediation of environmental pollutants and transforming them into economically viable products. Here, selective capturing of water or air pollutants is made possible through the simultaneous presence of functional nanomaterials as catalysts, such as nanoparticles or metal‐organic frameworks. While, delving into the inherent advantages of using nanofiber membranes such as flexible filtration properties, use of bio‐friendly materials, and stable filtration efficacy over longer durations. An approach across the disciplines, from materials sciences and chemistry to biology is needed. Hybrid electrospun nanofibers show great potential for capturing pollutants with the possibility of an integrated conversion. Utilizing the nanostructure for optimizing the capturing abilities as well as the conversion of these materials may provide a cost‐efficient and environmentally friendly way to get rid of unwanted pollutants. CO_2_ emissions might be tackled using multifunctional electrospun hybrid nanofibers as the fiber mats allow easy recycling and separation combined with flexible yet robust mechanical properties. Capturing and conversion of CO_2_ is just one of many applications in the field of environmental remediation where hybrid electrospun nanofibers can be useful. For instance, the removal of pollutants like heavy metal ions or dyes from water has already shown promising results and might be optimized in the future for widespread use. The biggest advantage of hybrid nanofibers in this field is multifunctionality offering filtration of the pollutants from air or water and the capability of conversion to more manageable or in some cases upcycled products as well. The exploitation of the potential of electrospinning nanofibers as a tool for environmental pollution remediation requires a deeper understanding and an efficient interface of nanostructure and chemistry.

Finally, the possibility of engineering the drug release mechanism based on controlled parameters has the potential to revolutionize drug dosing, delivery, and targeting. The potential of smart electrospun nanofibers could represent a significant advancement in theranostic applications, particularly in cancer and wound care. These theranostic systems can integrate treatment, monitoring, and diagnostic functions by efficiently encapsulating therapeutic and imaging agents separately within the nanofibers. For instance, delivering contrast agents and drugs simultaneously, providing the ability to perform theranostic study to image disease progression during treatment. Although electrospun smart hybrid nanofibers have yet to receive clinical approval for drug delivery and tissue engineering applications, most studies conducted to date have shown promising results in laboratory and animal testing settings.

There are some challenges as is with every advanced technology. The selection of materials and issues with the fabrication of hybrid nanofibers containing various constituents (e.g., polymers, ceramics, metals) can be complex due to different intrinsic material properties.^[^ ^218^ ^]^ Such challenges can often be overcome using co‐axial and multi‐nozzle electrospinning. Moreover, polymers, which are readily electrospinnable, are often dissolved in organic solvents, which puts some environmental cost, especially considering the levels required at industrial productions. Here, an emphasis on green solvents, water‐based polymer solutions, or deployment of techniques like melt electrospinning, could be one of the viable solutions.^[^ ^219^ ^]^ Additionally, the application of nanofiber across various fields such as healthcare and energy requires precise control over morphology and optical, mechanical, and electrical properties. However, the interplay of complex parameters introduces intricate changes that might not be replicated in every production cycle. Therefore, a deep level of understanding via accurate modeling and sophisticated control methods is necessary for the reproducible production of nanofibers.^[^ ^220^ ^]^ Another important challenge with traditional electrospinning has been the low production and adherence to strict quality control and safety regulations, which are demanded in the fields where nanofibers are generally implemented.^[^ ^221^ ^]^ Nevertheless, progress has been achieved steadily in the development of high throughput electrospinning via the implementation of techniques such as needless or multi‐needle electrospinning. Many start‐ups (Respilon, Elmarco, eSpin Technologies) have emerged focusing on volume productions or the manufacturing of sophisticated electrospinning machines, which enable the production of nanofibers at an industrial scale with precision and high quality. Consequently, offering products such as 3D Inserts, Bioweb, Healsmart, Nanospider, R‐shield that contains industrially produced nanofibers.^[^ ^222^ ^]^ Here, a multidisciplinary approach will undoubtedly help realize the full potential of smart electrospun hybrid nanofibers.

## Conflict of Interest

The authors declare no conflict of interest.
